# RNA integrity as a quality indicator during the first steps of RNP purifications : A comparison of yeast lysis methods

**DOI:** 10.1186/1471-2091-5-14

**Published:** 2004-10-01

**Authors:** Miguel López de Heredia, Ralf-Peter Jansen

**Affiliations:** 1 Gene Centre and Institute for Biochemistry, University of Munich, Feodor Lynen Str. 25, D-81377 Munich, Germany

## Abstract

**Background:**

The completion of several genome-sequencing projects has increased our need to assign functions to newly identified genes. The presence of a specific protein domain has been used as the determinant for suggesting a function for these new genes. In the case of proteins that are predicted to interact with mRNA, most RNAs bound by these proteins are still unknown. In yeast, several protocols for the identification of protein-protein interactions in high-throughput analyses have been developed during the last years leading to an increased understanding of cellular proteomics. If any of these protocols or similar approaches shall be used for the identification of mRNA-protein complexes, the integrity of mRNA is a critical factor.

**Results:**

We compared the effect of different lysis protocols on RNA integrity. We report dramatic differences in RNA stability depending on the method used for yeast cell lysis. Glass bead milling and French Press lead to degraded mRNAs even in the presence of RNase inhibitors. Thus, they are not suitable to purify intact mRNP complexes or to identify specific mRNAs bound to proteins.

**Conclusion:**

We suggest a novel protocol, grinding deep-frozen cells, for the preparation of protein extracts that contain intact RNAs, as lysis method for the purification of mRNA-protein complexes from yeast cells.

## Background

Genome analyses of a range of organisms have lead to the identification of an increasing number of putative RNA-binding proteins (RBPs) whose function is still unknown. RBPs have been found to act as integral part of ribonucleoparticles (RNPs) controlling gene expression at different levels [1]. RNPs are involved in controlling RNA export, RNA stability, RNA subcellular localization and mRNA translation [2]. It has been proposed that in this context RBPs could act as central coordinators in regulating the expression and fate of specific subsets of RNAs. This model is reminiscent of bacterial operons, where the expression of genes that act in the same pathway is regulated as one unit [3].

Recently, research has mainly been focused on identifying protein-protein interactions using two-hybrid interactions [4,5], immunopurification [6] or affinity purification [7]. So far, only few examples have been reported that were aimed at the identification of mRNA-protein interactions. In yeast, immunopurification has, for example, been used to enrich RNP complexes leading to the identification of 22 mRNAs localized to the bud tip [8], to the identification of Lhp1p associated mRNAs [9] and to the identification of mRNA export factor associated transcripts [10]. There are many examples for affinity purification methods in yeast, but perhaps the one that has been used most extensively is the Tandem Affinity Purification (TAP). TAP consists of two serial affinity purification steps of a protein tagged with a double epitope tag, without affecting the expression level of the protein. It was first described for identifying new protein components of the yeast U1 snRNP [11] and later used to identify protein-protein interactions in yeast [6], bacteria [12], *Trypanosoma brucei *[13], *Drosophila *[14] and mammals [15]. It has also been used to describe the set of mRNAs associated with the Puf family of RNA-binding proteins in yeast [16].

Besides the purification method, the way to lyse cells is also crucial. In yeast, different lysis methods are in use. Glass bead milling has been applied to identify RNAs from immunoprecipitated RNPs [9]. Both French Press and glass bead milling have been successfully used to characterize protein-protein [6,11] and protein-RNAs interactions [16]. However, the integrity of the mRNA has not been determined under the conditions used. Here, we show that existing lysis methods lead to extensive mRNA degradation even in the presence of RNase inhibitors. We also present evidence that a third method, grinding deep-frozen cells at ultra-low temperature, can be used to obtain intact mRNAs.

## Results

### Glass bead mill lysis leads to degraded RNAs

Breaking yeast with a glass bead mill is a common method to produce cell lysates. The principle is based on the physical rupture of the yeast's cell wall and cell due to the friction produced by glass beads rapidly moving through the cell suspension. One of the advantages of this method is the high lysis efficiency.

**Figure 1:**
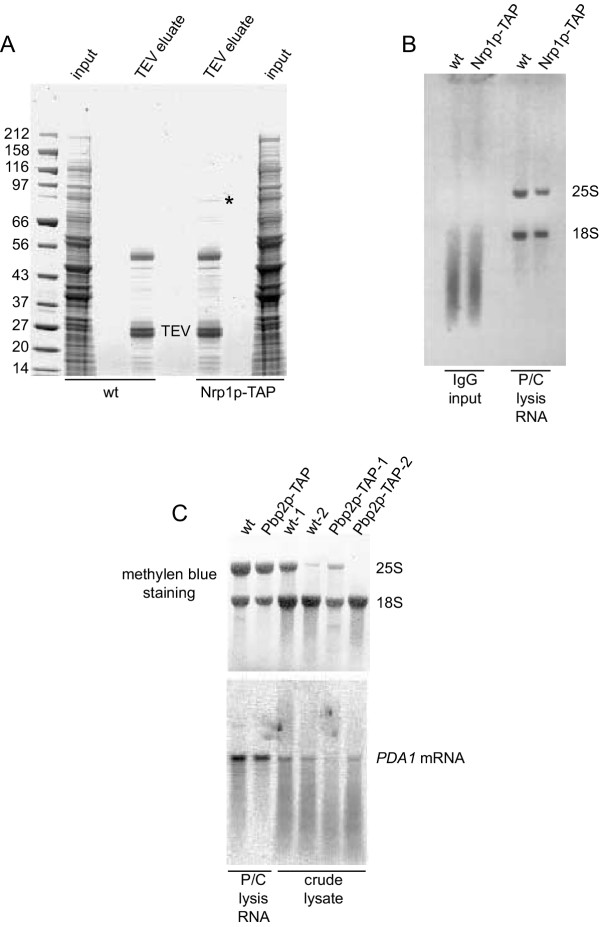
**TAP Purification of RNA-binding protein complexes leads to RNA degradation when cells are lysed in a glass bead mill. A**, 2 μl (corresponding to ~25 μg protein) of the input material for the IgG immunopurification (input) and the TCA-precipitated material from 200 μl of the TEV eluate from RJY358 (wt, untagged strain) and RJY929 (Nrp1p-TAP) were separated on a precast 4–12% gradient SDS polyacrylamide gel (Invitrogen) and stained with Coomassie. The molecular weight of the protein markers is indicated on the left. The band corresponding to the bait protein (Nrp1p-TAP) is labelled with an asterisk. **B**, 1 ml (corresponding to ~14 mg protein) of the input material for the IgG immunopurification (input) was phenol:chloroform extracted and 12 μg of the extracted RNA were separated on a 1.2% agarose-formaldehyde gel in the presence of ethidium bromide. As control for intact RNAs, 2 μg of total RNA (P/C lysis RNA) from the same strains prepared with a phenol method [18] were loaded in parallel as control. The position of the 18S and 25S ribosomal RNAs is indicated. **C**, crude lysate from strains RJY358 (wt, untagged) and RJY933 (Pbp2p-TAP) from two independent experiments were phenol extracted and 8 μg of the RNA were separated on a 1.2% agarose-formaldehyde gel and blotted onto a nylon membrane. 8 μg of total RNA (P/C lysis RNA) from the same strains prepared with a phenol extraction method [18] were loaded in parallel as control for intact RNAs. After methylene blue staining, the membrane was hybridized with a probe against *PDA1 *mRNA. The positions of the 18S and 25S ribosomal RNAs and the *PDA1 *mRNA hybridization signal are indicated.

We lysed two different yeast strains using a "bead-beater" bead mill in the presence of RNase inhibitors (100 U/ml SuperaseIn and 20 mM Ribonucleoside Vanadyl Complex, RVC) as described in Methods. We used a strain where Nrp1p, a putative RNA-binding protein that contains one RRM (RNA Recognition Motif) [17], has been tagged and a wild type strain. As shown in Figure 1A we could enrich the bait protein, Nrp1p, in the TEV eluate as compared to a purification from a control wild type strain performed in parallel. We then analyzed the RNA extracted from the input material at the IgG immunopurification step from both strains by agarose gel electrophoresis. The absence of 25S and 18S rRNAs in the extracts as compared to total RNA extracted by phenol [18] indicates that RNA was degraded (Figure 1B).

The chosen purification method involves several steps: lysis, extract preparation and IgG immunopurification. To rule out in which step RNA degradation takes place, we analysed mRNA integrity in samples taken at different purification steps (Figure 1C and data not shown). Therefore, we used a strain where Pbp2p, a putative RNA-binding protein that contains two KH-type 1 domains (hnRNP K homology domain) [19], has been tagged and a wild type strain. We found that extensive degradation already occurs during cell lysis (Figure 1C) as shown by Northern blot against a specific abundant mRNA (*PDA1*) or by direct staining of ribosomal RNAs after blotting with methylene blue (unbalanced ratio of 25S and 18S ribosomal RNAs in Figure 1C).

### Lysis by French Press leads to degraded RNAs

A second major lysis method for yeast cells is the French Press. Lysis occurs when the cell suspension is pressed through a small capillary. The pressure difference between the chamber and the capillary ruptures the cell.

**Figure 2:**
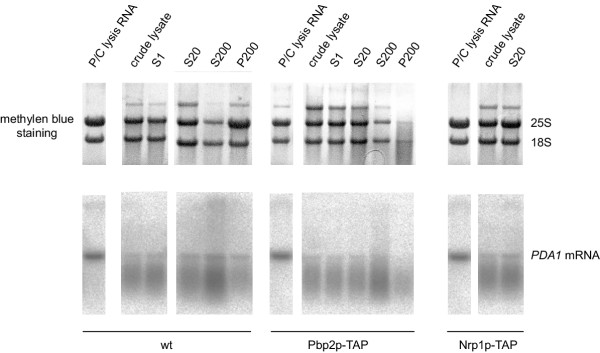
**RNA of cells lysed by French press is degraded. **Strains RJY358 (wt, untagged), RJY933 (Pbp2p-TAP) and RJY929 (Nrp1p-TAP) were lysed in a French Press and samples from crude lysate, supernatant of 1200 × g (S1), 20000 × g (S20), 200000 × g (S200) spins and pellet of 200000 × g (P200) spin were phenol extracted. 8 μg of the extracted RNA were loaded onto 1.2% agarose-formaldehyde gels and blotted onto nylon membranes. 8 μg of total RNA (P/C lysis RNA) from the same strains prepared with a phenol extraction method [18] were loaded in parallel as control for intact RNAs. After methylene blue staining, the membranes were hybridized with a probe against *PDA1 *mRNA. The positions of the 18S and 25S ribosomal RNAs in the methylene blue staining and the *PDA1 *mRNA hybridization signal are indicated. The nature of the third band that appears in the methylene blue staining on top of the 25S rRNA is unknown.

**Figure 3:**
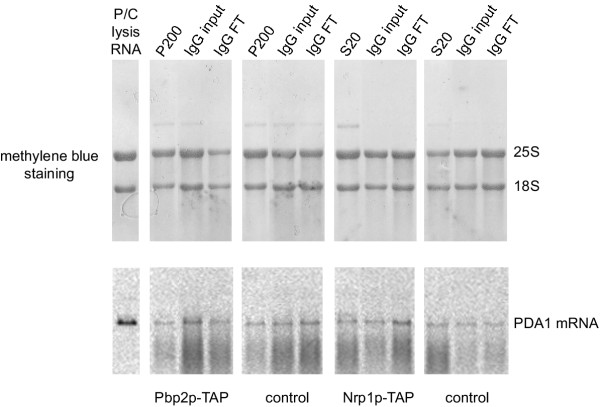
**RNA degradation is restricted to the lysis step prior to TAP purification. **Strains RJY933 (Pbp2p-TAP), RJY358 (wt, untagged strain) and RJY929 (Nrp1p-TAP) were lysed in a French Press and processed up to the IgG immunopurification as indicated in Methods. Samples from supernatant of 20000 × g (S20) spin and pellet of 200000 × g (P200) spin, as well as input material (IgG input) and flow through (IgG FT) from the IgG immunopurification step were phenol extracted. 8 μg of the extracted RNA were loaded onto 1.2% agarose-formaldehyde gels and blotted onto nylon membranes. 8 μg of total RNA (P/C lysis RNA) from strain RJY933 (Pbp2p-TAP) prepared with a phenol extraction method [18] were loaded in parallel as control for intact RNAs. The positions of the 18S and 25S ribosomal RNAs in the methylene blue staining and the *PDA1 *mRNA hybridization signal are indicated.

We lysed cells as described [11] in the presence of RNase inhibitors (100 U/ml SuperaseIn and 20 mM RVC), and spun the crude lysate at 1200, 20000 and 200000 × g as indicated in Methods. We then analyzed the RNA extracted from the different fractions by Northern blot. As shown in Figure 2, when analyzing RNA quality by methylen blue staining, RNA degradation is not as obvious as with bead mill lysis (compare the ratio between 25S and 18S ribosomal RNAs in Figure 2 with that in Figure 1C). However, all different strains tested (wild type, Nrp1p-TAP and Pbp2p-TAP) show a high degree of *PDA1 *mRNA degradation during cell lysis (crude lysate in Figure 2), although subsequent centrifugation steps do not further enhance degradation of this mRNA (S1, S20, S200 or P200 in Figure 2). We also tested, if the RNA becomes degraded during the first step of the TAP protocol. We took samples before and after the IgG immunopurification step and checked RNA integrity by Northern blot. As shown in Figure 3, no further degradation of the RNAs during this step can be detected. Taken together these results lead to the conclusion that most of the RNA degradation observed during French Press or glass bead mill lysis happens during cell rupture despite the presence of RNase inhibitors in the lysis buffer.

### Intact RNAs after lysis by mortar grinding

We also tested a third described method for cell lysis, which is grinding deep-frozen cells with a mortar [20-22]. This method breaks cells mechanically while keeping proteins and nucleic acids intact due to the lack of enzymatic activity at ultra-low breaking temperatures. After grinding, the extract is thawed in lysis buffer in the presence of RNase inhibitors (10 mM RVC and 100 U/ml SuperaseIn), immediately inactivating deleterious enzymatic activities.

**Figure 4:**
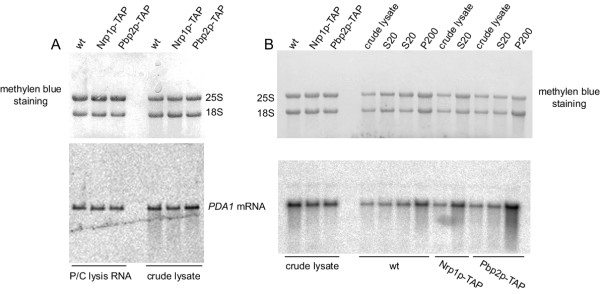
**RNA degradation is reduced during lysis by grinding. A**, Strains RJY358 (wt, untagged strain), RJY929 (Nrp1p-TAP) and RJY933 (Pbp2p-TAP) were manually ground in liquid nitrogen. Samples from crude lysate were phenol extracted and 8 μg of the extracted RNA were loaded onto 1.2% agarose-formaldehyde gels and blotted onto nylon membranes. 8 μg of total RNA (P/C lysis RNA) from strains RJY358 (wt, untagged strain), RJY929 (Nrp1p-TAP) and RJY933 (Pbp2p-TAP) prepared with a phenol extraction method [18] were loaded in parallel as control for intact RNAs. **B**, Strains RJY358 (wt, untagged strain), RJY929 (Nrp1p-TAP) and RJY933 (Pbp2p-TAP) were ground in a motor-driven mortar in the presence of dry ice. Samples from crude lysate, supernatant of 20000 × g (S20) spin, and pellet of 200000 × g (P200) spin were phenol extracted and 8 μg of the extracted RNA were loaded onto 1.2% agarose-formaldehyde gels and blotted onto nylon membranes. As control for RNA quality, RNA samples from independently lysed cells were loaded on the same gel. The positions of the 18S and 25S ribosomal RNAs in the methylene blue staining and the *PDA1 *mRNA hybridization signal are indicated.

First, we checked the integrity of the RNA after cells were lysed by manual grinding in liquid nitrogen. As shown in Figure 4A, RNA in crude lysate from the three strains tested (wild type, Nrp1p-TAP, Pbp2p-TAP) shows little difference from RNA isolated by direct phenol:chloroform extraction as indicated by the 25S/18S ribosomal RNA ratio and hybridization against *PDA1 *mRNA. We also tested RNA integrity during subsequent centrifugation steps after cell lysis with a motor-driven mortar. As shown in Figure 4B, the RNA from the different samples analyzed show only little degradation as judged by the *PDA1 *mRNA hybridization signal.

### Both RNase inhibitors are needed to keep mRNA intact

**Figure 5:**
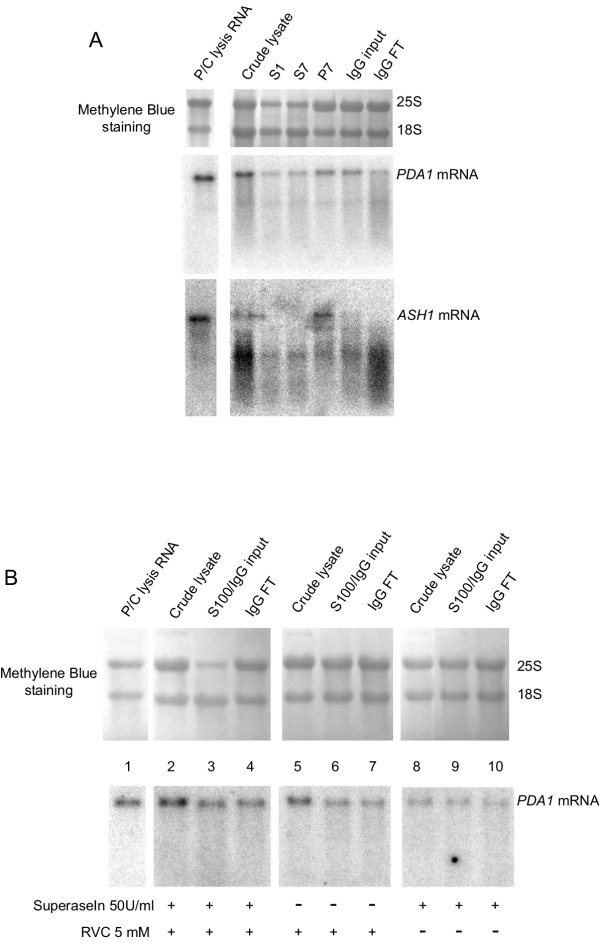
**Both RNase inhibitors are needed to keep the RNA intact when cells are lysed by mortar grinding. A**, Strain RJY1885 (She2p-TAP) was ground in a motor-driven mortar in the presence of dry ice. Samples from crude lysate and supernatants of 1200 × g (S1) and 7400 × g (S7) spins and pellet from 7400 × g spin (P7), as well as input material (IgG input) and flow through (IgG FT) from the IgG immunopurification, were phenol:chloroform extracted as indicated in Methods. 8 μg from the extracted RNA were loaded onto 1.2% agarose-formaldehyde gels. As RNA quality control, 8 μg of total RNA (P/C lysis RNA) extracted from the same strain with a phenol extraction method [18] were loaded in parallel. Membranes were first probed against *ASH1 *mRNA, stripped and then probed against *PDA1 *mRNA. The positions of the 25S and 18S ribosomal RNAs are shown, as well as the *PDA1 *and *ASH1 *mRNA hybridization signals. **B**, Sub2p-TAP strain [24] was ground in a motor-driven mortar in the presence of dry ice and thawed in lysis buffer supplemented with 50 U/ml of SuperaseIn and/or 5 mM RVC. Samples from crude lysate and supernatants of 100000 × g (S100) spin and flow through (IgG FT) from the IgG immunopurification, were phenol:chloroform extracted as indicated in Methods. 8 μg from the extracted RNA were loaded onto 1.2% agarose-formaldehyde gels. As RNA quality control, 8 μg of total RNA (P/C lysis RNA) extracted from the same strain with a phenol extraction method [18] were loaded in parallel. The positions of the 25S and 18S ribosomal RNAs are shown, as well as the *PDA1 *mRNA hybridization signal.

To optimize the lysis protocol, we also tested if both RNase inhibitors are needed to keep the RNA intact. For this purpose we first used a strain where She2p, an RNA-binding protein required for *ASH1 *mRNA localization, has been tagged [23]. She2p is known to bind to specific regions of *ASH1 *mRNA, which could help to protect the target mRNA. We lysed cells by mortar grinding and thawed them in lysis buffer with a final concentration of 170 U/ml of SuperaseIn as only RNase inhibitor. We took samples from crude lysate, different steps from the differential centrifugation and from the IgG immunopurification steps (input and flow through) and extracted the RNA. As shown in Figure 5A degradation of both *PDA1 *and *ASH1 *mRNAs is already detected in the crude lysate. Whereas no further degradation of *PDA1 *mRNA is detected during subsequent centrifugation, *ASH1 *mRNA is heavily degraded, indicating that there might not be a protective effect of RNA-binding proteins like She2p. In addition, when the extract was incubated with the IgG-coupled beads for 2 hours degradation becomes evident also for *PDA1 *mRNA (compare the last two lines in Figure 5A).

We then checked the effect of a reduced concentration of both RNase inhibitors, SuperaseIn and RVC, on RNA integrity. For this purpose we used a strain where Sub2p, an RNA-binding protein required for splicing and mRNA export, is tagged [24]. Sub2p is more abundant than She2p and believed to bind to mRNAs competent for export [25]. Like She2p, it could also have a protective function against mRNA degradation that could be more prominent due to its higher abundance. We lysed cells by mortar grinding and thawed them in lysis buffer containing either 5 mM RVC and 50 U/ml SuperaseIn, or 5 mM RVC, or 50 U/ml SuperaseIn. We took samples from crude lysate, supernatant of a 100000 × g spin, as well as input and flow through from the IgG immunopurification. As shown in Figure 5B, no degradation can be observed when RNA is stained with methylen blue and the signals from the 25S and 18S ribosomal RNAs are compared. In contrast, degradation of *PDA1 *mRNA is visible in crude lysates from samples that have been thawed in lysis buffers supplemented only with SuperaseIn as inhibitor (compare signal of RNA from lines 8 and 1 in Figure 5B), in concordance with previous results (Figure 5A). When RVC is present as sole RNase inhibitor (lines 5–7 in Figure 5B and compare with line 1), degradation is reduced. Only when both inhibitors are present in the lysis buffer (lines 2–4 in Figure 5B and compare with line 1) intact RNAs are detected.

## Discussion

Since most RNA-binding proteins fulfil their function in the context of RNA-protein complexes, knowledge of RNAs associated with specific RBPs is essential to elucidate their functions. In order to identify these transcripts, new methods must be developed or existing successful protocols for the identification of protein-protein interactions must be adapted. Although several recent publications have identified RNA partners from RNP-complexes [9,16], there are so far no reports on the quality of the RNAs purified from these complexes. Here we demonstrate that the method used for cell lysis of yeast cells is of great importance for isolation of intact complexes. Whereas standard lysis methods like disruption by French Press or glass bead mill lead to massive RNA degradation (Figures 1, 2, 3), grinding yeast cells at ultra-low temperatures leaves cellular RNA intact (Figures 4, 5).

The major difference between the lysis methods compared in this study is the timing between addition of the RNase inhibitor and target inactivation. In French Press and glass bead milling, a lag between addition and target inactivation occurs because inhibitors require cells to be broken in order to inactivate RNases as the yeast's cell wall acts as a barrier for large molecules. Since RNases are highly active enzymes, this could result in RNA degradation prior to their inactivation by the inhibitor. This lag could explain the degradation observed in samples from crude lysates produced by these lysis methods even in the presence of RNase inhibitors (Figures 1, 2, 3). This idea is supported by the lack of further degradation during subsequent steps (Figures 2, 3). Although we have only analyzed the first step of the TAP protocol, the IgG immunopurification, the conclusion from this experiment can be extrapolated to immunoprecipitated RNPs as the molecular interactions in both methods are equivalent.

In contrast to bead milling or disruption by French Press, the temperature during grinding is kept close to -80°C and enzymatic activities are essentially absent. The results from Figure 4 show only little RNA degradation after thawing ground extracts in buffer supplemented with RNase inhibitors, which supports cell grinding as an RNA integrity conserving method. Our data demonstrating enhanced stability of mRNAs using the grinding method are supported by previous successful purifications of snRNPs with intact snRNAs using grinding as lysis method [21,22].

RNA degradation can severely bias identification of RNA from purified RNPs because only RNA fragments that are protected from degradation would remain in the purified extract. Under these circumstances, the choice of the primer for retrotranscribing the pool of purified RNAs is critical since it is likely that only primers annealing close to or inside the protected sites would result in a cDNA product.

Furthermore, when such protected RNA fragments are used to probe micro-arrays, the type of the DNA-array also becomes crucial. Since many RBPs bind to the UTRs of mRNAs and many DNA-microarrays contain only DNA corresponding to the ORF of the genes, detection of bound RNAs by such microarrays could be biased against RBPs binding outside the ORF. This problem would be enhanced using arrays composed of long oligonucleotides since only RNAs whose protected regions overlap with the oligo sequence would be identified.

Another point to consider is the low abundance of several RNPs. As many RNA-binding proteins are expressed at low levels, purification of complexes using RNPs as bait might result in low yield of bait protein plus associated RNAs. Under these circumstances, it is mandatory to keep RNA intact at early stages (in the crude lysate), since the following purification steps might become an additional source of RNA degradation. This idea is also supported by our results (Figure 5A) where, even under optimised lysis conditions, mRNAs become sensitive to degradation during longer incubation times.

## Conclusions

Our data suggest that mechanical breakage of frozen yeast cells at ultra-low temperatures is the lysis method of choice for purification of intact mRNP complexes. Since in recent approaches [9,16] different lysis conditions have been used, a substantial number of protein-associated mRNAs might have been missed.

## Methods

### Yeast strains

Strains used in this work are isogenic with W303. The relevant genotypes are listed in Table 1.

**Table 1:** Strains used for this study. Strains used in this study and its relevant genotype. Modified gene is indicated in bold letters.

Strain	Genotype
RJY358	*MAT*a, *ade2-1*, *trp1-1*, *can1-100*, *leu2-3*, *112*, *his3-11*, *15*, *ura3*
RJY929	*MAT*a, *ade2-1*, *trp1-1*, *can1-100*, *leu2-3*, *112*, *his3-11*, *15*, *ura3*, ***NRP1*-TAP**::*k.l.TRP1*
RJY933	*MAT*a, *ade2-1*, *trp1-1*, *can1-100*, *leu2-3*, *112*, *his3-11*, *15*, *ura3*, ***PBP2*-TAP**::*k.l.TRP1*
RJY1885	*MAT*alpha, *ade2-1*, *trp1*::hisG, *can1-100*, *leu2-3*, *112*, *his3-11*, *15*, *ura3*, *pep4*::*LEU2*, *bar1*::*hisG*, ***SHE2*-TAP**::*k.l.TRP1*

TAP-tagged strains were generated by transformation with PCR products using the lithium acetate method [26]. The primers used for generating the PCR products from plasmid pBS1479 [11] are listed on Table 2.

**Table 2:** Oligonucleotides used in this study. Oligonucleotides used in this study for generating the tagged strains.

Oligo name	Sequence (5'-3')
Pbp2p-TAP forward	AATTGATAGATCAAATGCTGAACGTAAAAGAAGGTCGCCCCTCTCCATGGAAAAGAGAAG
Pbp2p-TAP reverse	GTAGTTTCTGTATTTTTATTTTCTATGTGTTTTTATTGACTAGTACGACTCACTATAGGG
Nrp1p-TAP forward	TAATAGCGCTTTCGGTAATGGTTTTAATAGTTCAATACGTTGGTCCATGGAAAAGAGAAG
Nrp1p-TAP reverse	AAATAAAAAATACAATGTGGTTGTGTGAAATTTATTGACCTCGTACGACTCACTATAGGG
She2p-TAP forward	GTTGTCGCTACTAAATGGCATGACAAATTTGGTAAATTGAAAAACTCCATGGAAAAGAGAAG
She2p-TAP reverse	GCTATTCATGTATATATATATGTTCTATTAACTAGTGGTACTTATTACGACTCACTATAGGG

### Lysis methods

Yeast cultures were grown at 30°C in YPD media [27] up to an OD_600 nm_/ml of 2–3. Cultures were harvested at 4°C and washed with ice-cold sterile water.

### Glass bead mill

Cell pellets from 10000 OD_600 nm _(5 l cultures with an OD_600 nm_/ml of 2) were harvested, washed with cold sterile water and immediately lysed or the wet pellet snap-frozen in liquid nitrogen. For lysis, harvested cells were resuspended in 30 ml lysis buffer (10 mM K-Hepes pH 7.9, 10 mM KCl, 1.5 mM MgCl_2_, 0.5 mM DTT, 1 μg/ml aprotinin, 0.8 μg/ml bestatin, 1 μg/ml leupeptin, 0.05 mg/ml pefabloc (Biomol, Germany), 0.7 μg/ml pepstatinA, 100 U/ml SuperaseIn (Ambion, UK), 20 mM Ribonucleoside Vanadyl Complex (RVC, NEB, UK)) and immediately broken. Frozen cells were stored at -80°C until used. Then, they were thawed in 30 ml lysis buffer and immediately lysed. Cells were lysed in a glass bead mill (Bead-beater, Biospec) in 3 cycles (3 min breaking and 5 min cooling). During lysis, cells were kept cold by a water-ice bath.

### French Press

Cell pellets from 5000 OD_600 nm _(2.5 l cultures with an OD_600 nm_/ml of 2) were harvested, washed with cold sterile water and immediately lysed or the wet pellet snap-frozen in liquid nitrogen. For lysis, harvested cells were resuspended in 15 ml lysis buffer (10 mM K-Hepes pH 7.9, 10 mM KCl, 1.5 mM MgCl_2_, 0.5 mM DTT, 1 μg/ml aprotinin, 0.8 μg/ml bestatin, 1 μg/ml leupeptin, 0.05 mg/ml pefabloc, 0.7 μg/ml pepstatinA, 100 U/ml SuperaseIn, 20 mM RVC) and immediately broken. Frozen cells were stored at -80°C until used. Then, they were thawed in 25 ml lysis buffer and immediately broken. Lysis was performed as described [11] with a cold chamber.

### Mortar grinding

Cell pellets corresponding to 3–15 l cultures, with an OD_600 nm_/ml of 2–3, were frozen by pressing the cell pellet through a 50 ml syringe directly into liquid nitrogen [20] and stored at -80°C until used. Up to 3 g frozen cells were ground manually in liquid nitrogen. Larger amounts were ground in a motor-driven mortar (Mortar Grinder RM 100, Retsch, Germany) for 15 min at pressure setting 5–7. Mortar and pestle were precooled twice with liquid nitrogen. Crushed dry ice was added continuously during grinding for keeping the mortar cold. Ground material was stored at -80°C until used. Typically, 25 g of ground cells were used for experiments running up to the first TAP immunopurification step, and 1–5 g of ground cells were used for experiments including only differential centrifugation steps. Ground cells were thawed in 1 ml of lysis buffer (40 mM K-Hepes pH 8, 1 mM MgCl_2_, 0.30% Igepal CA-630, 120 mM NaCl, 80 mM KCl, 2 mM EDTA, 1 mM DTT, 1 μg/ml aprotinin, 0.8 μg/ml bestatin, 1 μg/ml leupeptin, 0.05 mg/ml pefabloc, 0.7 μg/ml pepstatinA, 200 U/ml SuperaseIn, 40 mM RVC) per gram.

### Sample preparation

#### Glass bead mill and French Press

After cell lysis, cell extracts were subjected to serial centrifugations at 4°C. First, the crude lysate (CL) was spun 3 min at 1200 × g to pellet cell debris. The total extract (S1) was further spun for 20 min at 7500 × g and the supernatant collected (S7). Salt and buffer concentration were adjusted to IgG binding conditions (20 mM K-Hepes pH 7.4, 5 mM KCl, 0.75 mM MgCl_2_, 100 mM K-acetate, 10 mM Tris-HCl pH 8, 100 mM NaCl, 10 mM Mg-acetate, 1 mM EGTA, 0.1% Igepal CA-630, 0.5 mM DTT, 100 U/ml SuperaseIn, 10 mM RVC, 1 μg/ml aprotinin, 0.8 μg/ml bestatin, 1 μg/ml leupeptin, 0.05 mg/ml pefabloc, 0.7 μg/ml pepstatinA) with the addition of adjusting buffer (50 mM K-Hepes pH 7.4, 140 mM K-acetate, 30 mM Tris-HCl pH 8, 300 mM NaCl, 3 mM EGTA, 0.3% Igepal CA-630, 1 mM DTT, 100 U/ml SuperaseIn, 10 mM RVC, 2 μg/ml aprotinin, 1.6 μg/ml bestatin, 2 μg/ml leupeptin, 0.1 mg/ml pefabloc, 1.4 μg/ml pepstatinA). The resulting sample was again spun for 20 min at 20000 × g and the supernatant (S20) finally spun at 200000 × g for 20 min. The supernatant was collected (S200) and the resulting pellet (P200) was resuspended in 10 ml IgG binding buffer (20 mM K-Hepes pH 7.4, 5 mM KCl, 0.75 mM MgCl2, 100 mM K-acetate, 10 mM Tris-HCl pH 8, 100 mM NaCl, 10 mM Mg-acetate, 1 mM EGTA, 0.1% Igepal CA-630, 0.5 mM DTT, 100 U/ml SuperaseIn, 10 mM RVC, 1 μg/ml aprotinin, 0.8 μg/ml bestatin, 1 μg/ml leupeptin, 0.05 mg/ml pefabloc, 0.7 μg/ml pepstatinA).

#### Mortar grinding

After thawing the ground cells in lysis buffer, the sample was subjected to serial centrifugation at 4°C. First, the crude lysate (CL) was spun 3 min at 1200 × g to pellet cell debris. Total extract (S1) was further spun for 20 min at 7500 × g and the supernatant (S7) was collected. The resulting pellet (P7) was resuspended in 10 ml 0.5 × lysis buffer. Supernatant (S7) was again spun for 20 min at 20000 × g and the supernatant (S20) finally spun at 200000 × g for 20 min. The supernatant was collected (S200) and the resulting pellet (P200) was resuspended in 10 ml 0.5 × lysis buffer.

For TAP purifications of Sub2p, total extract (S1) was directly centrifuged at 100000 × g for 1 h (S100).

The lipid phase was discarded from all fractions.

100–300 μl aliquots from the different fractions were extracted immediately 3–4 times with phenol:chloroform:isoamylalcohol (pH 4), precipitated with sodium acetate and ethanol and stored at -80°C until analyzed.

Glycerol (5% final concentration) was then added to those samples used for further TAP purification steps. These samples were snap-frozen in liquid nitrogen and stored at -80°C for a maximum of 2–3 days. Aliquots were taken before freezing and immediately after thawing to control for degradation during freeze-thawing. These aliquots were extracted, precipitated and stored as described above. TAP purification was performed up to the IgG immunopurification step (first step of the TAP protocol) as described [11], using as IgG binding buffer 20 mM K-Hepes pH 7.4, 5 mM KCl, 0.75 mM MgCl_2_, 100 mM K-acetate, 10 mM Tris-HCl pH 8, 100 mM NaCl, 10 mM Mg-acetate, 1 mM EGTA, 0.1% Igepal CA-630, 0.5 mM DTT, 100 U/ml SuperaseIn, 10 mM RVC, 1 μg/ml aprotinin, 0.8 μg/ml bestatin, 1 μg/ml leupeptin, 0.05 mg/ml pefabloc, 0.7 μg/ml pepstatinA. Samples were taken before (IgG input) and after IgG immunopurification (flow through, IgG FT) and immediately extracted, precipitated and stored at -80°C as described above.

### Northern blot

Total RNA was isolated by direct phenol:chloroform extraction from the same strains used in the study as described [18], precipitated with sodium acetate and ethanol and kept at -80°C in ethanol until used as quality control. RNA pellets from the different samples were resuspended in DEPC-treated water, ratio A_260 nm_/A_280 nm _measured and equivalent amounts of RNA were loaded onto 1.2% agarose-formaldehyde gels. Samples loaded on gels for direct visualization were supplemented with 0.2 μg/μl of ethidium bromide in loading buffer. For Northern blot analysis gels were blotted onto nylon membranes, the membrane stained with methylene blue for determining transfer efficiency and probed against *ASH1 *and/or *PDA1 *mRNA (see Figure legend for details).

## List of abbreviations

The abbreviations used are: RNP, ribonucleoprotein; RBP, RNA-binding protein; TAP, tandem affinity purification; RVC, ribonucleoside vanadyl complex; RRM, RNA recognition motif; UTR, untranslated region; ORF, open reading frame; DEPC, diethylpyrocarbonate; TCA, trichloroacetic acid; TEV, tobacco etch virus protease.

## Authors' contributions

MLdH carried out the studies and drafted the manuscript. RPJ conceived of the study, and participated in its design and coordination. All authors read and approved the final manuscript.
